# Laser Powder Bed Fusion (LPBF) of In718 and the Impact of Pre-Heating at 500 and 1000 °C: Operando Study

**DOI:** 10.3390/ma14216683

**Published:** 2021-11-05

**Authors:** Asif Ur Rehman, Fatih Pitir, Metin Uymaz Salamci

**Affiliations:** 1 ERMAKSAN, Bursa 16065, Turkey; fatih.pitir@ermaksan.com.tr; 2 Department of Mechanical Engineering, Gazi University, Ankara 06570, Turkey; msalamci@gazi.edu.tr; 3 Additive Manufacturing Technologies Application and Research Center—EKTAM, Gazi University, Ankara 06560, Turkey; 4 Manufacturing Technologies Center of Excellence—URTEMM A.S., Ankara 06980, Turkey

**Keywords:** multiphysics, laser powder bed fusion, LPBF, temperature dependent properties, pre-heating, superalloy, In718

## Abstract

The morphology of a melt pool has a critical role in laser powder bed fusion (LPBF). Nevertheless, directly characterizing the melt pool during LPBF is incredibly hard. Here, we present the melt pool flow of the entire melt pool in 3D using mesoscopic simulation models. The physical processes occurring within the melt pool are pinpointed. The flow patterns throughout the same are exposed and measured. Moreover, the impact of pre-heating at 500 and 1000 °C has been described. The study findings offer insights into LPBF. The findings presented here are critical for comprehending the LPBF and directing the establishment of improved metrics for process parameters optimization.

## 1. Introduction

Additive manufacturing (AM) provides customized designs, reduced preparation time, and the ability to create complicated shapes. Many advanced technological applications [1] such as aerospace [2], biomedicine [3,4] and architecture [5,6] have considerable interest in it. Laser powder bed fusion (LPBF) is one of the most widely used additive manufacturing (AM) technologies because of its many advantages, which include significantly reduced structural limitations, high reproducibility, and on-time delivery [7]. In the LPBF, the metal particles are deposited layer upon layer via the blade or roller, followed by the fusing of particles by laser on particular locations to generate the desired slices, which is driven by CAD data [8]. There are a variety of flaws that are detrimental to efficiency and component quality [9,10,11] including balling, fractures, pores, and poor layer uniformity. As a result, it is necessary to better understand the deformations and the influence of input factors on the melt pool [12,13].

It has been shown that a variety of factors, including scan speed, laser power, particle sizes distribution (PSD), and layer height [14], have an impact on the melt pool and, therefore, the quality of the elements created [15,16]. Systematic attempts have been undertaken to explain the intricate melt pool dynamics [12,17,18,19,20], process parameters, and recurring defects in terms of the processing parameters that have an influence on the process. Studies by Hodge et al. and Lin et al. [21,22] looked at the effect of laser power and scan speed on the surface properties of LPBF parts. According to the research [21,22,23], irregularities, deformations, cracking, and other deficiencies on the surfaces are produced at a high scan rate, resulting in more surface defects. Studies have focused on the formation of the defect during the LPBF techniques of metallic metal powder [24]. The results of the investigation revealed that the energy density (ED) had a substantial influence on the formation of defects. The physics underpinning the dynamic interaction between the process parameters is, however, insufficiently explained by the available data. Concentrating only on the hit-and-trial strategy to extract the proper process variables throughout the LPBF experiments is both costly and time-consuming. Furthermore, the observational research [25,26] doubts the LPBF method’s ability to distinguish between the many complicated rules.

Melt stream inside the melt pool dictates heat hysteresis and mass flow in laser based 3D printing (e.g., powder bed fusion (PBF), laser metal deposition (LMD)) and therefore plays an essential part in melt pool formation [27,28], creation and progression of defects [29,30,31,32], solidification [33,34,35], and the formation of spatter [36,37,38]. As a consequence, the molten metal flow behavior has a significant impact on the surface morphology and mechanical characteristics of additively manufactured components [12,39,40,41,42]. As a result, monitoring transient characteristics during the melt is important for process optimization and also microstructure prediction models.

Given the difficulties of actually seeing the flow behavior within the melt pool, considerable scientific work has been conducted to investigate the flow dynamics under varying circumstances, i.e., with varying laser power or scan rates [43,44]. Experiments were also conducted to investigate the effect of a surface-active tracers on melt flow such as tungsten nanoparticles in LPBF of X-ray semitransparent Al based alloys. However, considering the intricacy of the underlying physics such as surface tension (surfactants itself can form high surface tension end), visualizing the associated multi-physics events is very difficult [27,45]. In fact, major visualization agents and parameters enhancements must be applied in most of the experiments [19,27,28,46,47]: certain implications are established for the purpose of visual convenience, some for the purpose of intricacy, whilst others due to the underpinning physics is not clear. Each of these inferences will have an effect on the reliability of the tests to some degree. As a consequence, despite the fact that the majority of the trials have been evaluated by evaluating the melt pool, the estimated melt flow outputs are often not accurate, and in certain cases are indeed contrary.

Flow prediction errors were discovered around the frontal depression [48,49], across the back [43,50], at the baseplate of the melt pool [51,52], over the depression’s exit [51,53], and the depression outflow [52]. Thus, comprehending the underlying melt flow characteristics inside the melt pool is essential in powder bed fusion [29,54]. The use of in-situ x-ray imaging has recently been shown for the investigation of laser additive manufacturing processes [28]. The latest research [27,55] utilized synchrotron x-ray scanning to track melt flow using tungsten particles, but only in single projection plane even though thousands of particles and tracers can be in the same plane. Given the complexity of the melt-pool in three dimensions the movement of the tracers, traced by the 2D imaging is put under question. The authors of this study recently developed the model for the flow in 3D [56,57].

Determining three-dimensional molten metal movement in PBF is complex, as the lasers scans faster, and the resultant melt pools are shallower. Owing to the difficulty, the full melt pool dynamics under LPBF settings has not really been exposed. Furthermore, part-scale thermal study shows interface heating continues to rise during deposition owing to heat buildup [58,59], and interfacial temperature fluctuation influences melt pool behavior. IR heating systems for layer pre-heating [60], scanner reheating as well as remelting methods [61,62,63], an additional unfocused laser beam [64], resistance heating of the base, [65,66] and inductive circuits on the baseplate [67], may lower the heat flow and is often used as an efficient thermal stresses reduction technique [68]. In LPBF, a substantial decrease in deformation could be seen for aluminum parts at a pre-heating degree of approximately 150 °C. Even with pre-heating level of 250 °C, distortions in the samples was no more detectable [69]. Kruth et al. [70] observed a 10 percent decrease in bending angles in the LPBF method by pre-heating up to 180 °C.

Part failure and deformation caused by residual stress may be avoided by pre-heating the chamber [65]. It was discovered that electron beam powder bed fusion (EPBF) part fabrication from pre-heated powders had an anisotropy in microstructure and mechanical properties [71]. Ali et al. showed significantly improved yielding and ductility in LPBF at 570 °C pre-heating temperatures [66]. Investigators also discovered that increasing the pre-heating temperatures allowed the breakdown of α′ martensitic in α + β stable microstructure. Recent research on the effect of pre-heating temperatures in metals printing has mostly focused on residual stress removal and deformation minimization, microstructure, and mechanical characteristics such as ductility as well as yield strength. Fully dense hard to weld materials such as alumina [72] and zirconia [64] were also printed using preheating of 800 and 1600 °C with LPBF respectively. Pre-heating can extend the range of LPBF from only weldable material processing to un-weldable materials such as ceramics. 

Aviation and turbines parts frequently utilize INCONEL 718 (IN718) [73]. The slow precipitation-hardening mechanisms of IN718 make it highly weldable (ideal for LPBF) [73]. The microstructure influences the mechanical characteristics of IN718 [74]. LPBF produces a consistent microstructure with little porosity and finer dendritic grain [74,75]. The regular dendritic structure disappears and a needle-like δ phase precipitates at grain boundaries when γ′ and γ″ phases dissolve in the matrix when heat treatment is applied A needle-like δ phase precipitates along grain boundaries whenever the γ′ and γ″ phases breakdown in the matrix during heat treatment, replacing the normal dendritic structure. [74]. Heat-treated LPBF has comparable tensile as well as ductility to wrought IN718 [74].

Using laser additive manufacturing, we developed a system for detecting melt flow behavior over the whole melt pool, which we describe in this article. We uncovered the melt flow behavior of the whole melt pool and investigated the driving factors of liquid flow as well as the fundamental processes throughout the melt pool. Finally, the impact of pre-heating temperatures on melt pool formation is investigated numerically. We provide an explanation for the track’s variable melt pool size and shape. Furthermore, the flow rates is used to describe the flow caused by the Marangoni effect and the resulting matching flow.

## 2. Materials and Methods

### 2.1. Powder Bed Modeling 

To simplify the process of computing the powder production and layering process, this will be divided into two stages: in the beginning, a multitude of particles are dropped directly on the surface to generate a powder stack; afterwards, the blade/recoater moves over the surface at a set speed and the particles advance into build chamber to create the layer.

The interaction technique using the nonlinear elastic equation is employed to assess the elastic real contact force [76], and the damping criterion is theoretically used to recognize the dissipation of mechanical energy [49,77,78].

It is at this point in the perpendicular planes when natural contact forces and damping forces in elastic materials occur. Relative stiffness across the plane is perpendicular, and the Young’s modulus as well as mass of the plane are both constant. In order to account for elastic contact force, no micro-slip method is used in the tangential route [76]. Table 1 shows the PSD for ERMAK-A241-IN718 supplied by ERMAKSAN, Bursa, Turkey, with D10, D50, and D90. The particle have been simulated utilizing the PSD that has been provided.

Throughout the whole study, the discrete element modeling (DEM) module from Flow Science, Santa Fe, NM, USA, has been employed to simulate the layer-by-layer deposition of SS316L stainless metal powder. A layer of powder was deposited utilizing discrete microparticles, rather than viewing the powder layer as a plate of uniform size.

**Figure 1:**
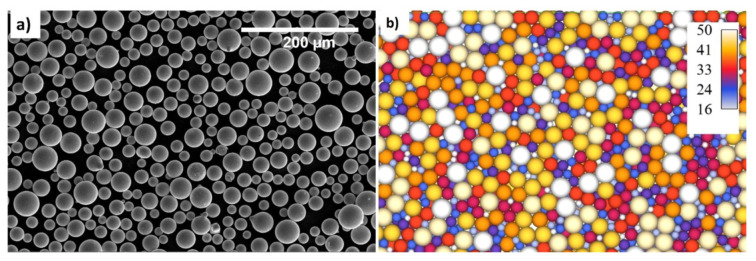
(**a**) Powder particle SEM and (**b**) discrete element modeling.

The powder particles are seen clearly in Figure 1. According to the SEM image in Figure 1a, the real powder particle may be interpreted as round in a reasonable approximation. The particle sizes were known to be consistent with the experiment computation, and the particle sizes were found to be consistent with D10, D50, and D90 in the appropriate proportions (see Figure 1). Figure 1b depicts the particle that was created using the simulation.

### 2.2. Modeling of Powder Bed Deposition Process

**Figure 2:**
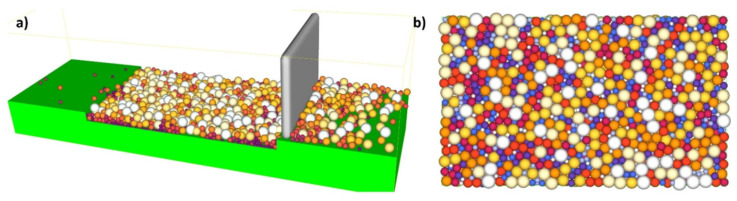
(**a**) Powder bed deposition; (**b**) deposited layer.

Primarily based on the theoretical model mentioned above, the ERMAK-A241-IN718 The modeling of the metal powder creation process was carried out. Layer thickness is 50 microns due to the powder particles level. Deposition process is shown in detail in the second illustration. Simulation of DEM during powder bed deposition is shown in Figure 2a. Figure 2b shows a 50-micron layer that has been deposited. In the DEM model (see Figure 2a, a 100-micron layer was deposited. It was found that when the spaces on the outer/free surface were left in place, the layer of powder had a packing density of 65 percent; however, when the gaps on the outer/free surface were eliminated, this density rose to 90 percent.

### 2.3. Modeling of Thermophysical Properties

**Figure 3:**
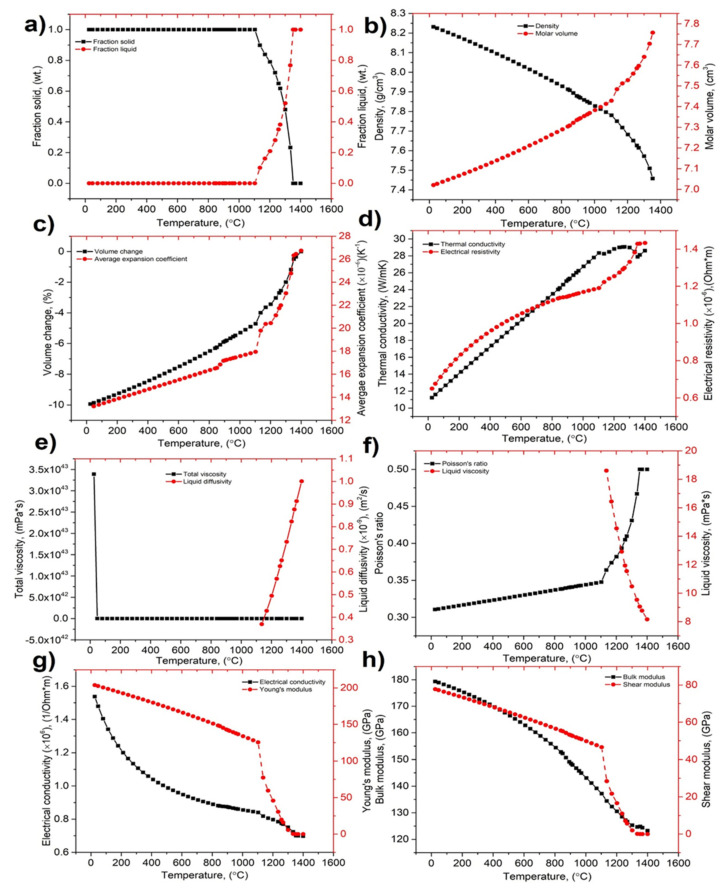
Temperature-dependent properties: (**a**) solid state to liquid state, (**b**) density and molar volume, (**c**) average expansion coefficient and volume change, (**d**) electrical resistivity and thermal conductivity, (**e**) total viscosity and liquid diffusivity, (**f**) Poisson’s ratio and liquid viscosity, (**g**) electrical conductivity and young’s modulus, and (**h**) bulk modulus and shear modulus.

Temperature related physical properties of ERMAK-A241-IN718 have been simulated utilizing Sente Software, UK, depending on the chemical composition (Table 2) of ERMAK-A241-IN718 obtained from ERMAKSAN, Bursa, Turkey, as shown in Figure 3.

The super quick melting as well as solidification that occurs throughout the LPBF process has an implication on all of the thermo-physical characteristics involved. The cornerstone of the LPBF process modeling of the metal powder is a complete material characteristics simulation.

In order to simulate the thermo-physical behavior of the ERMAK-A241-In718, we used the chemical composition specified in Table 2 of this same document. The results are shown in Figure 3. As demonstrated in the graphs in Figure 3, melting as well as solidification may have an effect on the average expansion coefficient but also density [79]. Figure 3a shows the change in solid state to liquid state with temperature, Figure 3b shows the change in density and molar volume with temperature, and Figure 3c shows the average expansion coefficient and volume change with temperature, Figure 3d shows the electrical resistivity and thermal conductivity with temperature. Similarly, Figure 3e shows total viscosity and liquid diffusivity with temperature, and Figure 3f shows Poisson’s ratio and liquid viscosity with temperature.

Because of this, although the density decreases consistently with heating, the shift in averaged expansion coefficient is not totally consistent, which is one of the most important influencing elements during the treatment with the laser irradiation. The decrease in surface tension may also be shown to be not totally continuous, which results in a non-uniform Marangoni flow characteristic. The Marangoni flow develops in the fusion zone as a result of the difference in surface tension between the heated and cooled ends [36], which causes the flow to be unstable. It is also possible to illustrate that the change in Poisson’s ratio and Youngs moduli is completely non-consistent. While the latent heat increases with temperature in a constant manner, the increase in conductivity does not, which is one of the most important influencing elements for heat dissipation inside the building platform [79].

### 2.4. Numerical Model

A computational fluid dynamics (CFD) framework was developed and implemented by including particular subprocesses from the FLOW-3D 11.2v CFD package as well as weld module of Flow Science, Inc., United States of America (USA). Some equations have been explained below.

Multiple parameters and assumptions are considered in the research for simplicity: (1) the melting within the melt stream is assumed incompressible Newtonian; (2) the changes in mass attributable only to metal evaporation are often not included.

In Equations (1)–(3), the corresponding equations that can be solved for mass continuity, momentum conservation, as well as energy conservation are shown, respectively:

(1) $$\nabla \cdot \vec{v}=0$$

(2) $$\frac{\partial \vec{v}}{\partial t}+\left(\vec{v}\cdot \nabla \right)\vec{v}=-\frac{1}{\rho }\nabla \vec{P}+\mu \nabla^{2}\vec{v}+\vec{g}\left[1-\alpha \left(T-T_{m}\right)\right]g\left[1-\alpha \left(T-T_{m}\right)\right]$$

(3) $$\frac{\partial h}{\partial t}+\left(\vec{v}\cdot \nabla \right)h=\frac{1}{\rho }\left(\nabla \cdot k\nabla T\right)$$

where v denotes the velocity profile. $\vec{P}$ denotes pressure, μ denotes viscosity, and, $\vec{g}$ denotes gravity function, h denotes specific enthalpy, ρ specifies density, and k denotes heat conductivity Volume of fluid (VOF) models employ the free surface for data acquisition [80]. A simple Equation (4) may be used to explain the VOF approach.

(4) $$\frac{\partial V_{F}}{\partial t}+\nabla \left(\vec{\nu }\cdot V_{F}\right)=0$$

where $V_{F}$ denotes the volume fraction of metal present within the cell. $V_{F}$ = 1, implies that the cell is completely filled with fluid, while $V_{F}$ = 0 indicates that the cell is completely devoid of fluid. Counts in the middle demonstrate that there is free space on this cell’s entire surface.

Variability in melt pools may be caused by a variety of factors, including thermophysical properties, vapor suppression, as well as penetration. Moreover, because the Rosenthal approach is re-extracted out from heat equation thus removes evaporation, convection, and even the Marangoni influence, the heat equation may be written as [81,82], the corresponding term in Equation (5) for melt pool diameter extracted from Rosenthal formula [83] to explain the role played by thermo-physical characteristics in melting pool heterogeneity in heat transfer [81]:

(5) $$\omega =\sqrt{\frac{8}{\pi e}\cdot \frac{P\eta }{\rho C_{p}V\left(T_{m}-T_{0}\right)}}$$

where ω is width of the melt pool, P defines beam power, and η specifies absorptivity, $C_{p}$ specifies heat capacity, *V* specifies scanning velocity, the melting temperature is specified by $T_{m}$, and the preheating level is specified by $T_{0}$. According to the assumption of thermally independent physical qualities as well as the thermophysical conductivity utilized to determine the size of the melt pool, the Rosenthal solution is found.

Additionally taken into consideration are the effects of recoil pressure, as well as vapor suppression, on the melt pool scale [84]. Equation (6) might be used to determine each individual recoil pressure:

(6) $$P_{S}=A\cdot \exp\left\{B\left(1-\frac{T_{V}}{T}\right)\right\}$$

the primary coefficient A can be calculated by using the following: A = β$P_{0}$, β ∈ [0.54, 0.56]. $P_{0}$ is the atmospheric pressure. The secondary coefficient *B* can be computed using the following equation: in which the *B* = $\Delta H_{v}$/$RT_{v}$, $\Delta H_{v}$ stands for the vaporization heat and the gas constant (R). $T_{v}$ stands for the saturation temperature [84].

It is well established that the energy density of the beam follows a Gaussian distribution. Throughout scanning, the laser moves at a constant scan speed, and the ED of the laser may be described mathematically [84] as the following Equation (7):

(7) $$q=\frac{2Ap}{\pi R_{b}^{2}}exp\left[-2\frac{{\left(x-\nu t-x_{0}\right)}^{2}+{\left(y-y_{0}\right)}^{2}}{R_{b}^{2}}\right]$$

where *A* defines the absorption of its laser beam by the powder particles, p represents laser power, *R_b_* denotes the radius of the laser, and v indicates the pace of scanning. ($x_{0}$,$\;y_{0}$) is the coordinate system. The starting position of the laser beam center [84] is represented by this value. The beam radius has been represented by $R_{b}$. Convection and radiation were resolved on this free surface, but evaporation could not be disregarded on the molten pool’s surface because of the presence of water. It follows as a consequence that the energy equation for the melt pool’s surface can be written as an Equation (8) [84], which is as follows:

(8) $$\frac{\partial T}{\partial \vec{n}}=q-h_{C}\left(T^{1}-T_{0}^{1}\right)-\sigma_{0}\varepsilon \left(T^{4}-T_{0}^{4}\right)-q_{evap}$$

where, $h_{c}$ denotes the coefficient of convective heat transfer, $T_{0}$ is the ambient temperature, and the Stefan–Boltzmann constant is denoted by $\sigma_{0}$. ε denotes the emissivity of a material, and $q_{evap}$ is the heat transfer owing to evaporation, and it may be expressed [84] by the Equation (9) which reads as follows:

(9) $$q_{evap}=\omega_{0}L_{v}=\exp\left(2.52+6.121-18,836T-0.5logT\right)L_{v}$$

where, $\omega_{0}$ denotes the evaporation rate and, $L_{v}$ denotes the latent heat of evaporation. In this study, a new equation was constructed explicitly for mass flow rate, as specified and determined by Equation (10) in the findings and discussion section.

(10) $$\dot{m}=\int \rho \cdot \vec{v}d\vec{A}$$

### 2.5. Configuration of the Modeling Environment and Variables

Primarily In718 powder generation and deposition process was mainly modeled utilizing the above-mentioned theoretical framework. The material properties of In718 for the LPBF simulations given below were selected from the Flow-3D available material dataset. The bed layer size was kept constant at 100 μm. The power (200 W), speed (3 m/s) and spot size has been kept constant. The preheating temperature has been changed from ambient temperature to 500 and 1000 °C in three cases. The domain size has been provided in the figure within the Supplementary Data.

Mobile workstation Precision 7530 form Dell Inc. Round Rock, TX, USA, with Windows 10 Pro for Workstations 64-bit was used for the simulations. It has a processor Intel(R) Xeon(R) E-2186M (CPU @ 2.90 GHz (12 CPUs), ~2.9 GHz) with a memory of 32,768 MB RAM and available OS Memory of 32,524 MB RAM. A simulation can take from 24 h to 1 week to complete.

### 2.6. Experimental Procedure

To validate the mathematical method, single-track LPBF tests were also carried out. The singular melting track was produced using an ERMAKSAN 250, which employs a fiber laser manufactured by ERMAKSAN EON Photonics, Bursa, Turkey. Atomized ERMAK-A241-In718 powder with an essentially spherical shape was utilized in the experiments. The beam’s diameter has been kept at 85 μm. The laser speed and power has been kept constant as described in the simulation.

**Figure 4:**
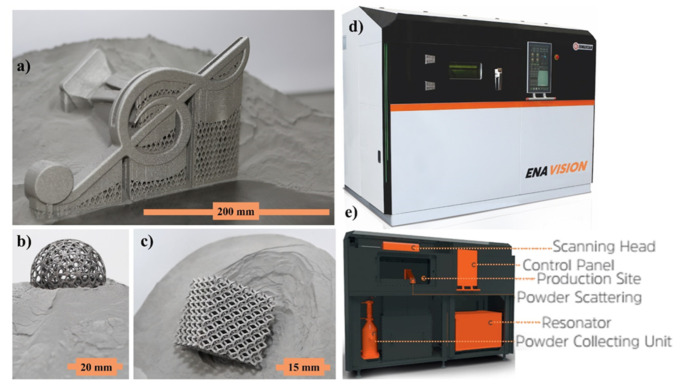
(**a**) Faucet design, (**b**) ball within cage design, (**c**) lattice structure manufactured specimens, (**d**) ENAVISION 250 used for validation, (**e**) machine breakdown.

Figure 4a–c shows a printed specimen. Figure 4d depicts the ENAVISION 250 LPBF system that was used for validation. The equipment breakdown is shown in Figure 4e. General specifications for ENAVISION 250 LPBF system have been provided in Table 3.

The OM micrographs were taken using an ZEISS smartzoom digital microscope from Carl Zeiss Microscopy Deutschland GmbH, Germany was used to capture optical micrographs.

## 3. Results and Discussion

**Figure 5:**
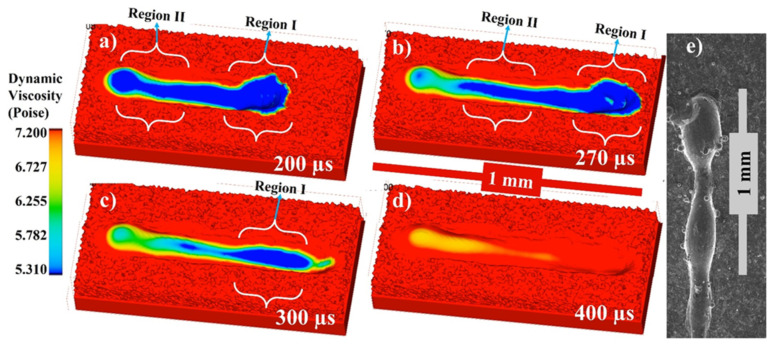
Single track dynamic viscosity profile at (**a**) 200, (**b**) 270, (**c**) 300, (**d**) 400 µs and (**e**) shows the experimental comparison through the SEM of the irradiated single track.

Figure 5a–d shows the melt pool dynamic viscosity at 200, 270, 300, and 400 µs, respectively, and Figure 5e shows the experimental comparison through the SEM of the irradiated single track.

When the powder material is exposed to heat (laser) of high intensity and is heated far beyond the melting point and even to the boiling point. A recoiling pressure is applied to the melt pool due to vaporization surface, which results in a depression zone (as shown in region I). While heat transfers from the outside to the interior of the material below via the depression zone, it also melts the layer below as well. The melt flow characteristics of LPBF form quite differently, attributable to the multifaceted underlying physics which will be discussed in detail in the following sections. Due to the formation of the depression zone the melted metal is pushed backwards as shown in the region II. This is one of the influencing factors in the melt pool dynamics apart from the others which will be discussed in the following sections.

When the powder material that is exposed to heat (laser) of high intensity and is heated far beyond the melting point and even to the boiling point. A recoiling pressure is applied to the melt pool due to vaporization surface, which results in a depression zone. While heat transfers from the outside to the interior of the material below via the depression zone, it also melts the material’s inner area as well. The melt flow characteristics of LPBF form quite differently, attributable to the multifaceted underlying physics.

**Figure 6:**
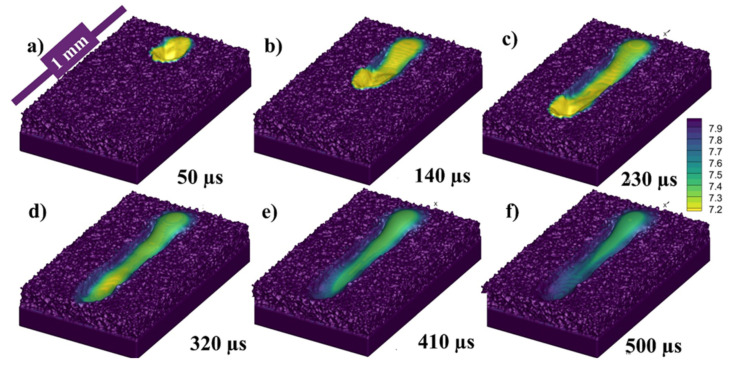
Single track melt pool profile at (**a**) 50, (**b**) 140, (**c**) 230, (**d**) 320 (**e**) 410 and (**f**) 500 µs.

Figure 6a–f presents the state of the melt pool at 50, 140, 230, 320, 410, and 500 µs, respectively, with respect to the density of the material. Because the depression zone is constantly heated, the flow from higher temperatures fluid (with lower density) continues to flow. However, as the melted metal has a greater temperature (low density) than those at the top of the melt pool, it rises up owing to buoyancy throughout backward transmission and creates a vortex at the rear edge of the melt pool, as illustrated. To make things easier to explain, the density color gradient has been presented in Figure 6.

The surface tension difference is one of the another most influencing factor in the liquid state. When the surface tension difference is generated in the liquid between the two ends of the liquid, a very strong pull force is produced from the high surface tension end to the low surface tension end called the Marangoni force.

The flow streams will be discussed in detail in the following discussion.

**Figure 7:**
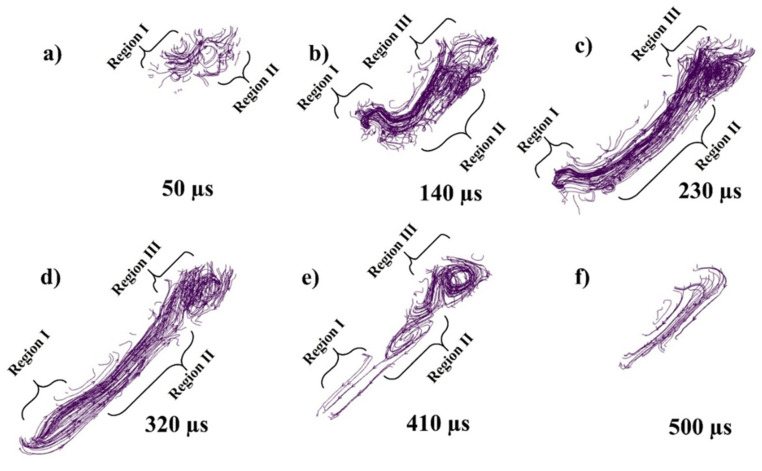
Stream traces of single-track flow at (**a**) 50, (**b**) 140, (**c**) 230, (**d**) 320, (**e**) 410, (**f**) 500 µs.

While Figure 7a–f presents the flow stream traces of the melt pool flow at 50, 140, 230, 320, 410, and 500 µs, respectively. When the laser starts to irradiate the powder, each powder particle starts to melt and joins the melt pool formation, melting is highly dependent on the energy density and particle size distribution (PSD), all this happens in the matter of micro-seconds as shown in Figure 7a. The region I shows the irradiating particle being melted by laser while a Marangoni flow is generated from top (high temperature end) to bottom (low temperature end) as shown in region II of Figure 7a. As the laser moves a small amount of the melted pool forms a flow in the forward direction contributed by both the Marangoni force (with the powder particle in front of the laser)and the recoil pressure as shown in Figure 7b region I. Similarly, a large amount of fluid is pulled backwards due to the strong Marangoni force and the recoil pressure as shown in Figure 7b region II. At the rear end of the melt pool a circular flow or swirl is formed due to the high surface tension at the edges of the melt pool as well as the rear end.

As the flow progresses with the movement of the irradiating laser, the recoil pressure and the Marangoni force keep contributing to pushing the flow backwards. The surface tension difference triggered by the temperature difference (between the rear end and the laser irradiation end) pulls the liquid to the rear as shown in Figure 7c,d. As the single track progresses the flow contributed by the nonuniform cooling of the track can form distorted circular flows or multiple swirls, which contribute more to the uneven width and height of the irradiated single track.

**Figure 8:**
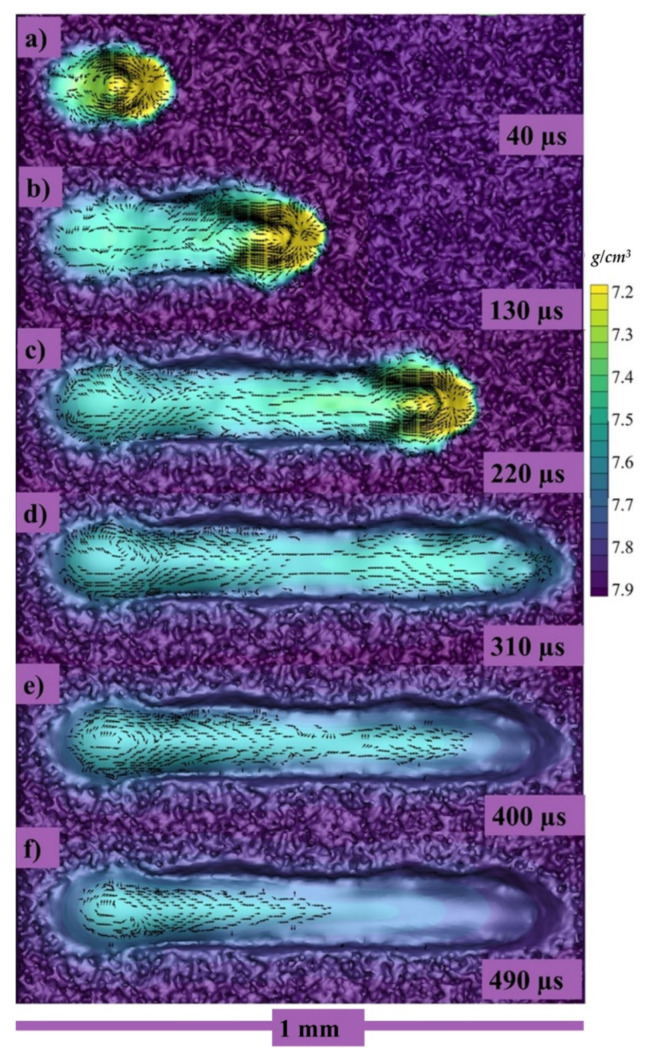
Longitudinal velocity vectors of single track at (**a**) 40, (**b**) 130, (**c**) 220, (**d**) 310, (**e**) 400, (**f**) 490 µs.

The top view of the same single track can be seen in Figure 8a–f at 40, 130, 220, 310, 400, and 490 µs, respectively, where the color gradient is showing the density of the melt pool.

**Figure 9:**
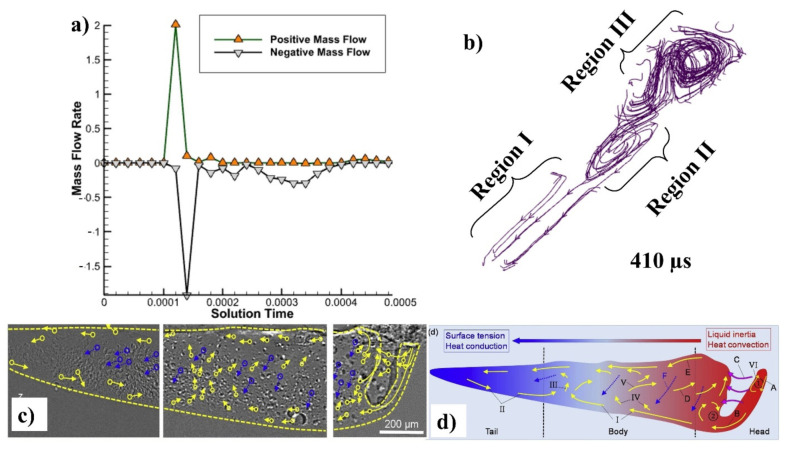
(**a**) Mass flow rate forward and backwards, (**b**) flow streams at 410 µs, (**c**) X-ray synchrotron imaging of melt pool [27], and (**d**) schematics of melt pool from the same [27].

A 2D cross section of the track was obtained, and the amount of liquified metal going forward as well as backward was computed using mass flow rate calculations in order to fully comprehend the mass flow rate, as shown in Figure 9a. It depicts the flow dynamics of the melt pool prior and afterwards laser irradiation. The positive mass flow rate indicates that the flow is moving forward, whereas the negative mass flow rate indicates that the flow is moving backward. A small portion of the melt pool flows forward until the laser is irradiated, but when the laser passes over that area, the melt pool is dragged backwards owing to the difference in surface tension between the two regions. X-ray synchrotron imaging of melt pool in LPBF of Al based alloys using tungsten nanoparticle markers can be seen in Figure 9c, and schematics of melt pool from the mentioned experiments can be seen in Figure 9d.

The melt pool is propelled by five main factors. the Marangoni force, which is also known as Bernoulli’s principle, impels a fluid in one direction, from the elevated temperatures zone to the reduced temperature region for such a material having a negative coefficient of surface tension (all metals and alloys, with the exception of a few special ones, will experience a negative temperature coefficient of surface tension in the range from low surface tension area to high surface tension region) [54,85,86,87,88]. Recoil pressure is exerted toward the vaporized surface imposed as a result of an inward force perpendicular to the vaporized surface [89,90]. For very fast vapor plumes (which may reach speeds of 110^2^–10^3^ m s^−1^ [91]), friction between the liquid and gas phases produces shear stress at the interfaces [86,92]. Hydraulic pressure is capable of exchanging energy through pressure (hydrostatic force) or momentum (hydrodynamic force) [93]. Liquid is propelled down a density gradient by buoyancy force [43,93,94].

**Figure 10:**
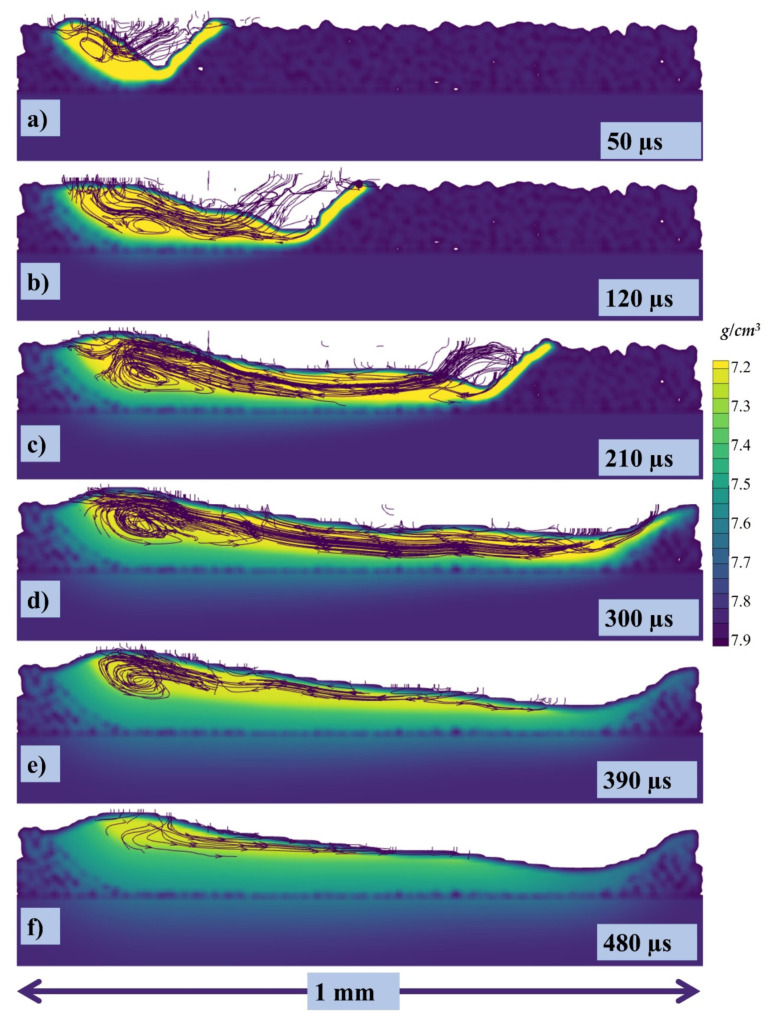
Stream traces of the cross-section at (**a**) 50, (**b**) 120, (**c**) 210, (**d**) 300, (**e**) 390, (**f**) 480 µs.

The cross section in the middle of the melt pool in lateral direction has been taken with stream traces and can be seen in Figure 10a at 50, 120, 210, 300, 390, and 480 µs, respectively, where the color gradient is showing the density of the melt pool. When the laser starts to irradiate the powder particle start to form the melt pool and with the movement of the laser the pool is pushed backwards due to the recoil and Marangoni force as shown in Figure 10a where the stream traces show the path of the melt pool from its initial point in the powder layer. Similarly, when the laser keeps moving further the flow keeps being pulled from the higher surface tension (or relatively lower temperature region) as shown in Figure 10b, and a circular flow pattern starts to appear. The pull force toward the rear end and the circular flow pattern keep on increasing as shown in Figure 10c,d. As the whole single track starts to solidify and cool down, a hump is generated which contributes to the uneven height of the melt pool. The higher concentration of the melt pool causes the rear end to cool down slowly as compared to the region with the lower concentration as seen in Figure 10d. Now, at the end of the single-track cooling, when the melt pool concentrated region has higher temperature and the less concentrated has lower temperature, the liquid metal starts to flow in the opposite direction and this also provides evidence that the Marangoni is the dominating force.

**Figure 11:**
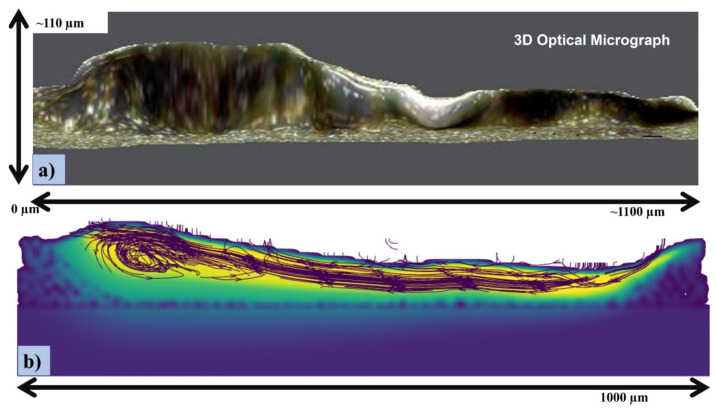
(**a**) OM of the single track; (**b**) stream traces of the cross-section at 300.

Figure 11 shows the experimental comparison in lateral direction through the OM of the irradiated single track. It is in good agreement with the simulation results where the recoil pressure and Marangoni force is causing irregular height of the melt pool.

**Figure 12:**
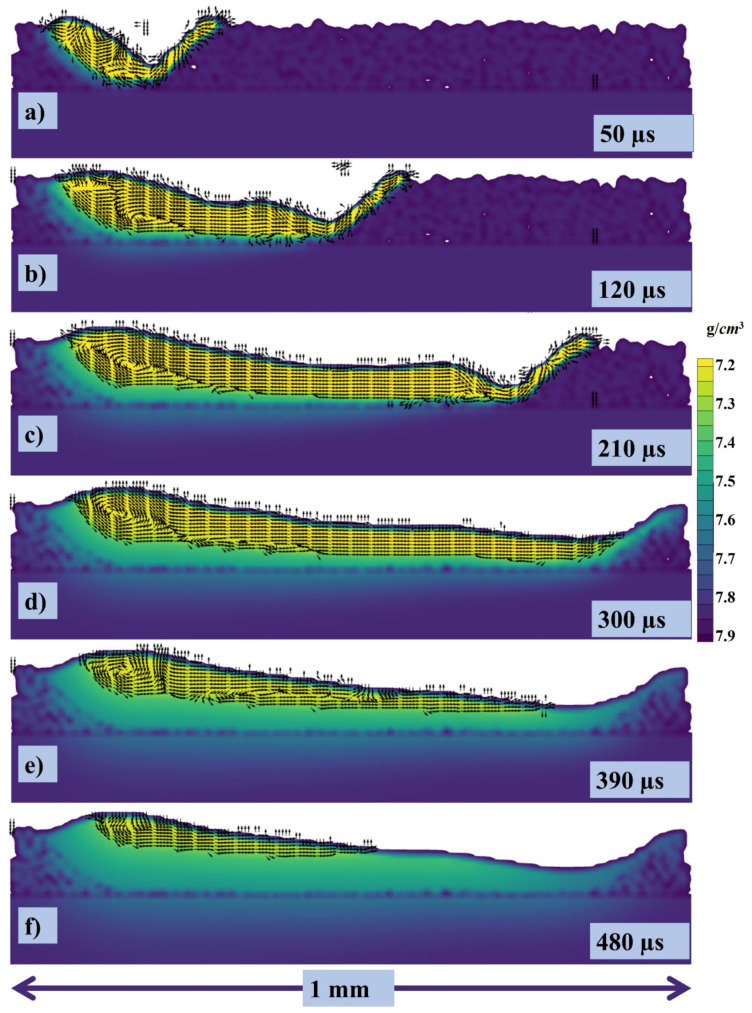
Velocity vectors of the cross section at (**a**) 50, (**b**) 120, (**c**) 210, (**d**) 300, (**e**) 390, (**f**) 480 µs.

The cross section has been taken with velocity vectors of each point within the melt pool and can be seen in Figure 12a–f at 50, 120, 210, 300, 390, 480 µs, respectively.

**Figure 13:**
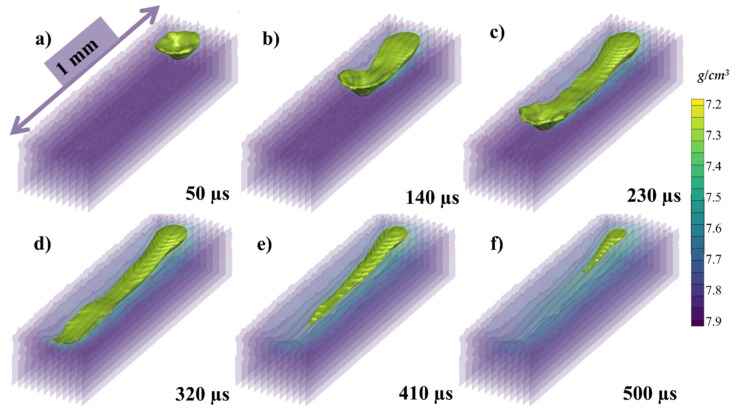
The isometric view of the melt pool (**a**) 50, (**b**) 120, (**c**) 230, (**d**) 320, (**e**) 410, (**f**) 500 µs.

The isometric view of the melt pool has been taken without the solid powder (transparent region) and can be seen in Figure 13a–f at 50, 120, 230, 320, 410, and 500 µs, respectively, where the color gradient in the transparent region is again showing the density of the melt pool. Here, the solidification of the melt pool can be elaboratively described. When the laser starts to irradiate a depression is formed as shown in Figure 13a and the recoil pressure and Marangoni pushes the melt pool backwards as shown in Figure 13b, and the surface tension furthers the pull as shown in Figure 13c. After the laser irradiation has been finished the dominating or the only force that is contributing to the flow is Marangoni force as shown in Figure 13d–f.

Because of the strong vaporization that causes the depression zone, the flow profile in a depression-mode melt pool is very complicated. The flow profile around depression [the area under laser] is dominated by vaporization-related pressures (i.e., recoil pressure and vapor plumes drag) and the Marangoni force. With Marangoni force as well as the vapor plumes friction, the flow across the front portion of the depression flows upwards [52,90,95], whereas the flow towards the depression’s base flows downward owing to recoil pressure, as shown in both transverse views as well as the crosswise view in Figure 7b,c and Figure 8a–c. The flow bypassing the depression zone is primarily driven by the hydraulic differential pressure, as a highly pressurized area forms in the fluid flow front of the depression zone but the front depression wall continues to “push” the front liquid throughout its path. It is worth noting that the upward flows may momentarily vanish or increase, as a result of the interaction between the downwards recoil pressures and the upward vapor plumes friction, as well as the Marangoni forces at front depression wall [86,90,92] as shown in Figure 7b,c, Figure 8a–c and Figure 10a–c.

As the depression region progresses, the low hydraulic-pressure area develops behind the depression’s base [96], It causes drawing the adjoining fluid into the rear side of the depression zone. As for the melt, its surface flows to rear end from an elevated temperature area to a relatively low temperature (Marangoni force), emulating huge mass transfer. The stream going backwards picks up momentum as it travels backwards.

The flow keeps on flowing from elevated temperatures fluid (with lower density) because it is being continuously heated at the depression zone. However, the melted metal rises up due to buoyancy throughout backward transmission because it has higher temperature (low density) than those at the top of the melt pool and it forms a vortex at the rear side of the melt pool as shown in Figure 7b–d, Figure 10b–d and Figure 12b–d.

Apart from an upward motion, the stream in the medium level of the melt pool has a strong propensity to travel forward, drawn by the low hydraulic pressure area surrounding the depression zone caused by the high-speed flows under Bernoulli’s effect. Backward flow on the surface is produced by Marangoni forces until the “rear end” area of the melt pool, whereas the flow from the rear end starts to flow forward from the base of the rear end due to formation of vortex. When the flow comes back just at melt pool middle, it collides with the flow coming from the depression zone, momentum is transferred forming a joint flow upward and later forming two vortexes. During the collision a rear end vortex and the frontal vortex is formed.

The flows from transverse view create two more closed loops, which are likewise governed by Marangoni force (consistent with recent research [54]), following a similar process to that of the longitudinal view. The flow produces a clockwise vortex 1 front of the beam as well as a counterclockwise vortex 2 after the laser beam has irradiated, as shown in the images. The frames per micro-second in video format for (1) cross sectional stream traces at RT, (2) cross sections velocity vectors at RT, (3) velocity vectors isometric view at RT, and (4) velocity vectors top view at RT, can be obtained from Supplementary Data.

Pre-Heating at 500 °C

**Figure 14:**
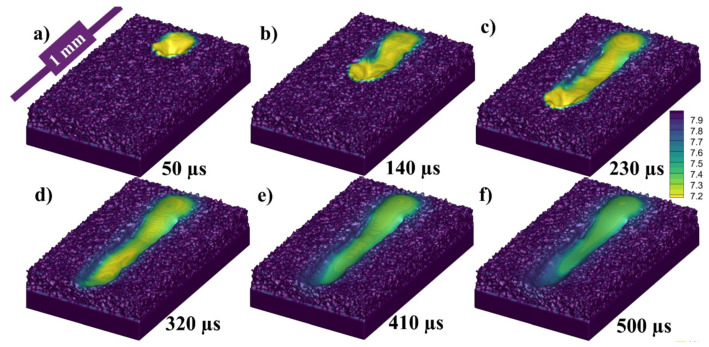
Single track melt pool profile at (**a**) 50, (**b**) 140, (**c**) 230, (**d**) 320 (**e**) 410 and (**f**) 500 µs.

The state of the melt pool in Figure 14a–f at 50, 140, 230, 320 410 and 500 µs, respectively, with 500 °C pre-heating has been shown, similar to the Figure 6 at the room temperature. The difference from the one at RT can be clearly visualized in terms of melt pool and solidification in this figure while the change in flow will be discussed in the following sections. Similar to the melt pool irradiation at the room temperature, the density decreases rapidly with melting due to the change in state which in turn increases the volume of the fluid slightly. However, as the temperature of the surrounding is kept at 500 °C the solidification is much slower, even after 500 µs there is still the melt pool remaining.

**Figure 15:**
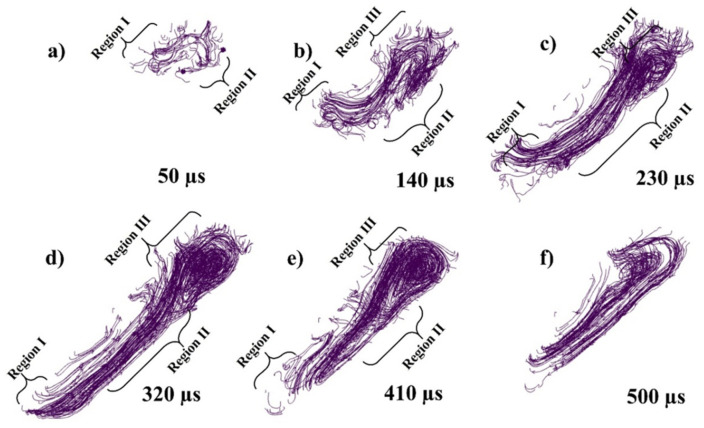
Stream traces of single track in 3D at (**a**) 50, (**b**) 140, (**c**) 230, (**d**) 320 (**e**) 410 and (**f**) 500 µs.

Figure 15a–f presents the flow stream traces of the melt pool flow at 50, 140, 230, 320, 410, 500 µs, respectively, with 500 °C pre-heating. When the laser starts to irradiate the powder, the particles start to melt more quickly and joins the melt pool formation quicker than in RT. The region I shows the irradiating particle being melted by laser while a Marangoni flow is generated from top (high temperature end) to bottom (low temperature end) as shown in region II of Figure 15a which is stronger than the one RT. As the laser moves a small amount of the melt pool is formed in the forward direction as shown in Figure 15b region I, which is more stable when compared with the one at RT. Similarly, a large amount of fluid is pulled backwards as shown in Figure 15b region II and a circular flow or swirl is formed due to the high surface tension at the edges of the melt pool as well as the rear end. The melt pool becomes wider as compared with the one RT.

As the flow progresses with the movement of the irradiating laser, the recoil pressure and the Marangoni force keep contributing to pushing the flow backwards as shown in Figure 15c,d. However, in Figure 15e,f, a positive aspect of pre-heating can be visualized when compared with Figure 6 at RT, that there has not been the distortion in the flow pattern because of the slow solidification.

**Figure 16:**
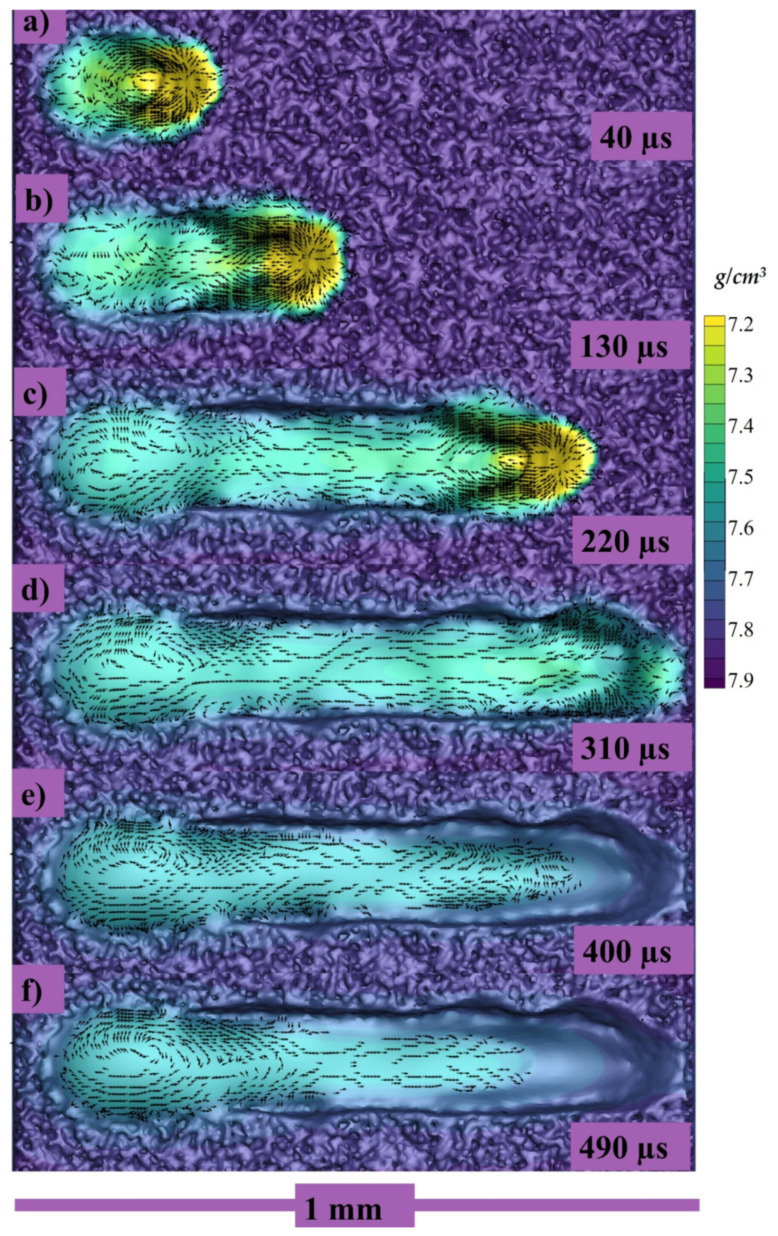
Longitudinal velocity vectors of single track at (**a**) 40, (**b**) 130, (**c**) 220, (**d**) 310, (**e**) 400, (**f**) 490 µs.

The top view of the same single track can be seen in Figure 16a–f at 40, 130, 220, 310, 400, and 490 µs, respectively, where the color gradient is showing the density of the melt pool.

**Figure 17:**
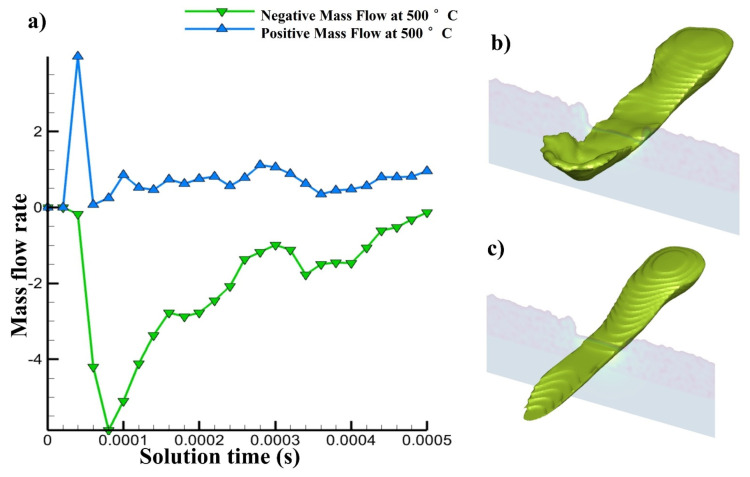
(**a**) Mass flow rate forward and backwards, (**b**,**c**) melt pool passing from cross section at different time.

A 2D cross section was taken in the single track with 500 °C pre-heating, the melt pool flowing forward and backward was calculated in Figure 17a through mass flow rate calculation to understand the mass flow rate like the one in Figure 9a. In this case, the laser irradiation at 500 °C pre-heating causes the melt pool to flow differently than it did before. The positive flowrate indicates forward movement, whereas the negative indicates backward flow. After being exposed to laser radiation, some of the melt pool flows forward; however, when the laser passes across that location, some of the melt pool is dragged backwards. However, it keeps moving forward after the laser irradiation as shown in the graph, even though it also keeps moving backwards. The cross section through which the mass flow has been described can be seen in the Figure 17b,c.

**Figure 18:**
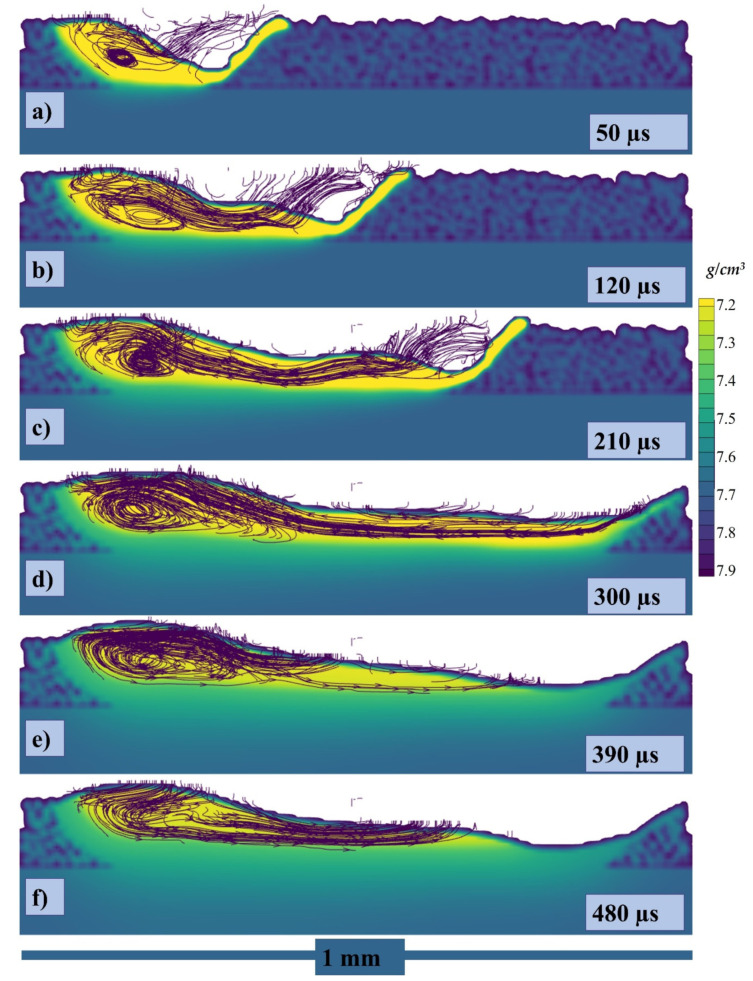
Stream traces of the cross-section at (**a**) 50, (**b**) 120, (**c**) 210, (**d**) 300, (**e**) 390, (**f**) 480 µs.

The cross section in the middle of the melt pool with 500 °C pre-heating has been shown in Figure 18a–f at 50, 120, 210, 300, 390, and 480 µs, respectively, where the color gradient is showing the density of the melt pool. Due to the chamber heating the density of the powder particle is slightly decreased. When the laser starts to irradiate the powder the melt pool is formed more rapidly as compared with the one at RT as shown in Figure 18a, where the stream traces show the path of the melt pool from its initial point in the powder layer. Similarly, when the laser keeps moving further the flow keeps being pulled from the higher surface tension as shown in Figure 18b and a circular flow pattern starts at the rear end. The melt pool is deeper due to the pre heating and the circular flow pattern is wider as shown in Figure 18c,d. The higher concentration of the melt pool causes the rear end to cool down slowly as compared to the region with the lower concentration, the liquid metal starts to flow in the opposite as shown in Figure 18e,f. Due to the pre-heating the melt pool has more time to settle down, as the flow moves backwards in Figure 18e,f and the height of the hump is decreased.

**Figure 19:**
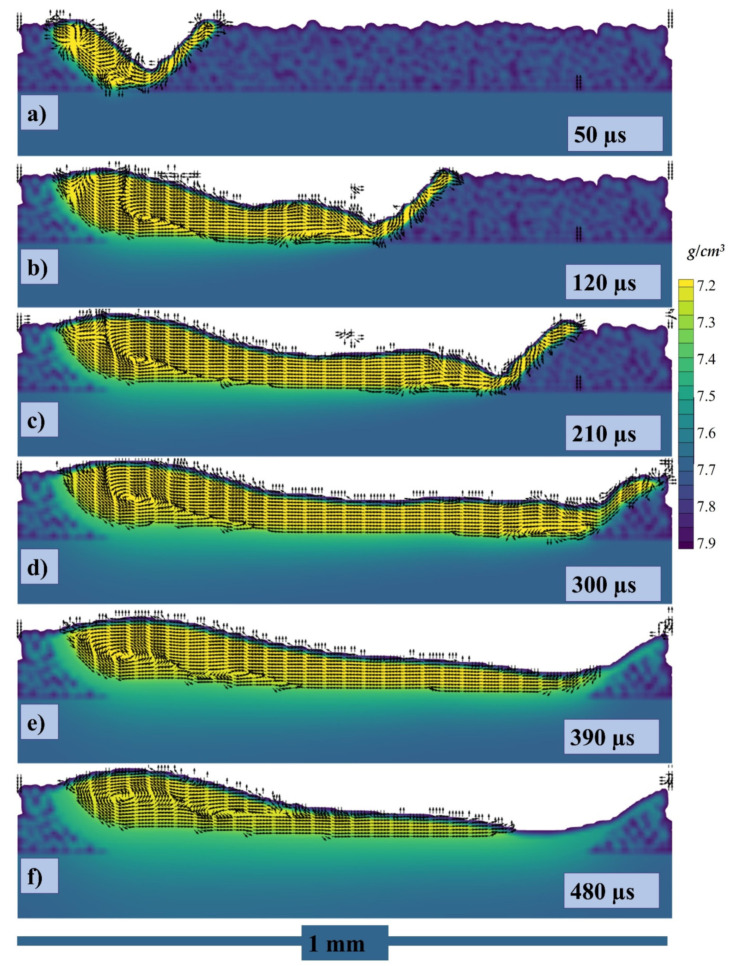
Velocity vectors of the cross section at (**a**) 50, (**b**) 120, (**c**) 210, (**d**) 300, (**e**) 390, (**f**) 480 µs.

The cross section with 500 °C pre-heating has been taken with velocity vectors of each point within the melt pool and can be seen in Figure 19a–f at 50, 120, 210, 300, 390, 480 µs, respectively. Similar to the one discusses above, when the laser starts to irradiate 500 °C pre-heated chamber the powder particle starts to form the melt pool more rapidly as shown in Figure 19a. A reaction force in the deeper depression zone is generated on the laser contacting surface as shown by the velocity vectors in the top surface in Figure 19a similar to the one in RT. However, as the laser progresses, due to the surrounding air the upper region of the melt pool which is in direct contact with the air starts to cool (can be visualized with the velocity vectors on the tope surface caused by Marangoni force as well as by the density color gradient) with huge increase in the surface tension, in Figure 19b–d.

A circular flow pattern can also be visualized with the velocity vectors at the rear end of the melt pool in Figure 19b–f. However, as the front-end temperature decreases with time and the surface tension over there increase, it starts to pull the liquid in the opposite direction as seen in Figure 19e,f from the bottom of circular flow pattern.

**Figure 20:**
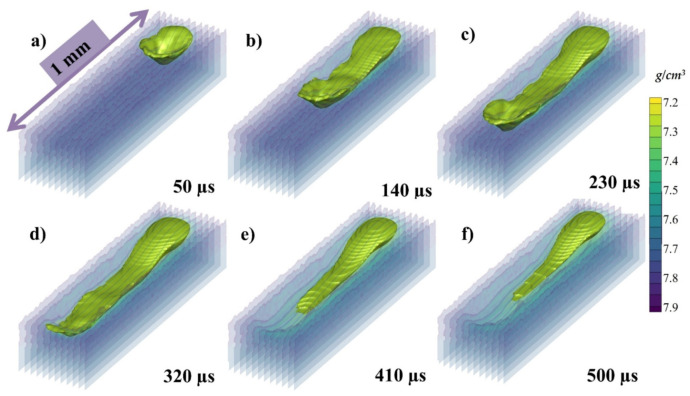
The isometric view of the melt pool with 500 °C pre-heating at (**a**) 50, (**b**) 120, (**c**) 230, (**d**) 320, (**e**) 410, (**f**) 500 µs.

The isometric view of the melt pool with 500 °C pre-heating has been taken without the solid powder (transparent region) and can be seen in Figure 20a–f at 50, 120, 230, 320, 410, and 500 µs, respectively, similar to the one at 500 °C pre-heating in Figure 13. Here, the melt pool flow can be visualized which is wider and deeper with the same energy density. When the laser starts to irradiate a depression is formed as shown in Figure 20a and the recoil pressure and Marangoni pushes the melt pool backwards as shown in Figure 20b, and the surface tension furthers the pull as shown in Figure 20c. After the laser irradiation has been finished the dominating or the only force that is contributing to the flow is Marangoni force as shown in Figure 20d–f. The frames per micro-second in video format for (1) cross sectional stream traces at 500 °C, (2) cross sections velocity vectors at 500 °C, (3) velocity vectors isometric view at 500 °C, and (4) velocity vectors top view at 500 °C, can be obtained from Supplementary Data.

Pre-Heating at 1000 °C

**Figure 21:**
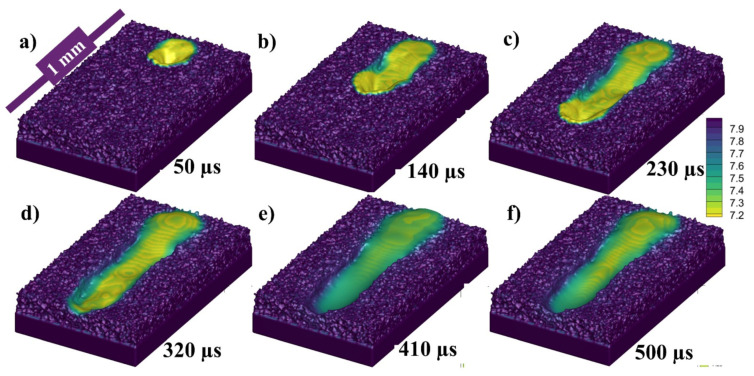
Single track melt pool profile with 1000 °C pre-heating at (**a**) 50, (**b**) 140, (**c**) 230, (**d**) 320 (**e**) 410 and (**f**) 500 µs.

The state of the melt pool in Figure 21a–f at 50, 140, 230, 320, 410 and 500 µs, respectively, with 1000 °C pre-heating, similar to the Figure 14 and Figure 6 at the 500 °C pre-heating and RT. The difference from the previous ones can be clearly visualized in terms of melt pool size and in this figure. The width and depth of the melt pool has been significantly increases and solidification at the end of the 500 µs has not completed and much of the melt pool in in liquid state.

**Figure 22:**
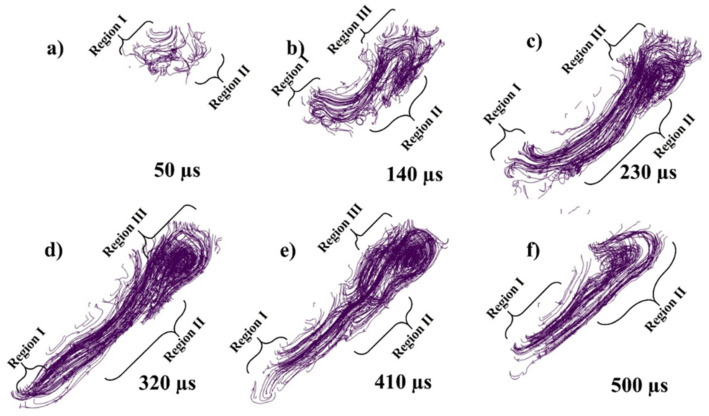
Streamtraces of single track in 3D at (**a**) 50, (**b**) 140, (**c**) 230, (**d**) 320 (**e**) 410 and (**f**) 500 µs.

Figure 22a–f presents the flow stream traces of the melt pool flow at 50, 140, 230, 320, 410, 500 µs with 1000 °C pre-heating. As the powder bed is preheated at 1000 °C, the laser needs little energy to melt, the particles form the melt pool more rapidly. The region I shows the irradiating particle being melted by laser while a Marangoni flow is generated from top (high temperature end) to bottom (low temperature end) as shown in region II of Figure 22a which is stronger than the one at 500 °C pre-heating. As the laser moves a small amount of the melt pool is formed in the forward direction as shown in Figure 22b region I (it is more significant from 500 °C pre-heating as well as RT). Similarly, a large amount of fluid is pulled backwards as shown in Figure 22b region II and a circular flow or swirl is formed due to the high surface tension at the edges of the melt pool as well as the rear end. The melt pool becomes wider as compared with the one 500 °C pre-heating.

As the flow progresses with the movement of the irradiating laser, the recoil pressure and the Marangoni force keep contributing to pushing the flow backwards as shown in Figure 22c,d. However, in Figure 22e,f, a positive aspect of pre-heating can be visualized when compared with Figure 15 at RT, that there has not been the distortion in the flow pattern because of the slow solidification. Secondly, another positive aspect at 1000 °C pre-heating is that the melt pool has time to flow back towards the front end, in Figure 22e,f, when the front-end temperature drops, and the rear end temperature is high due to the accumulated melt pool.

**Figure 23:**
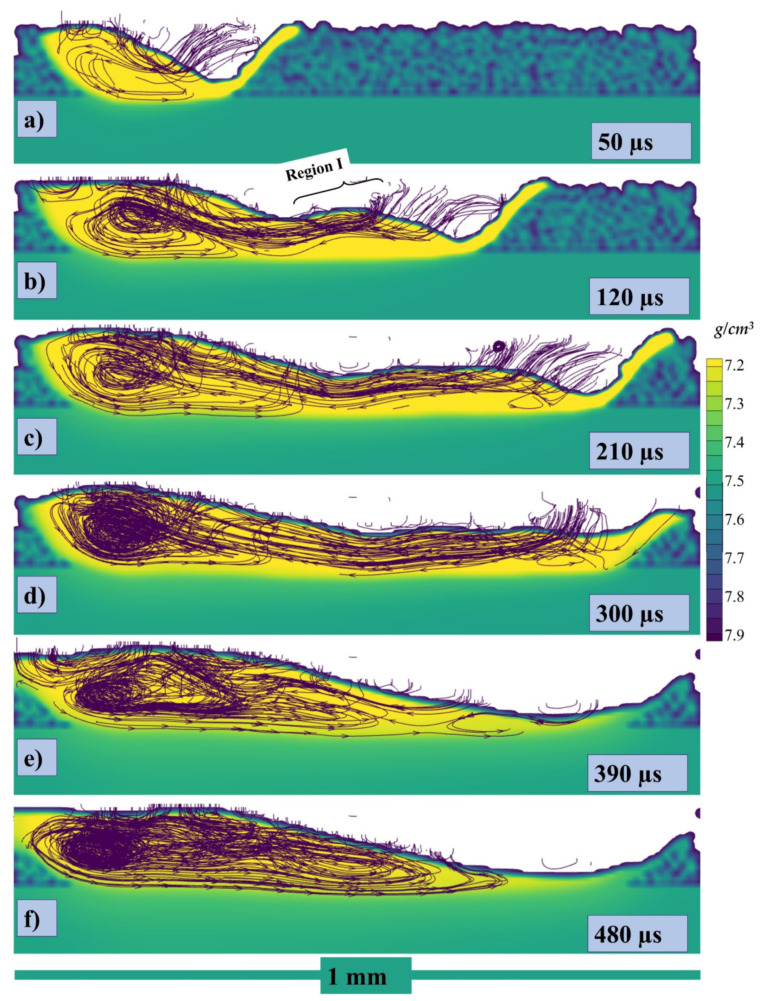
Stream traces of the cross-section with 1000 °C pre-heating at (**a**) 50, (**b**) 120, (**c**) 210, (**d**) 300, (**e**) 390, (**f**) 480 µs.

The cross section in the middle of the melt pool with 1000 °C pre-heating has been shown in Figure 23a–f at 50, 120, 210, 300, 390, and 480 µs. Due to the pre heating the density of the powder particle and the baseplate is decreased as seen by the color gradient in comparison to previous. When the laser starts to irradiate the powder melt pool is formed easily and significantly bigger as shown in Figure 23a, where the stream traces show the path of the melt pool from its initial point in the powder layer. Similarly, when the laser keeps moving a recoil pressure reaction force pushes the liquid backwards and upward simultaneously as shown in ‘region I’ in Figure 23b, the flow keeps being pulled due to Marangoni, and a circular flow pattern starts at the rear end which is more significant in comparison. The melt pool is deeper due to the pre-heating and the circular flow pattern is wider as shown in Figure 23c,d. However, the hump is less significant when compared with the previous ones, because the surface tension difference around the melt pool is low consequent of the pre-heating. The higher concentration of the melt pool causes the rear end to cool down slowly as compared to the region with the lower concentration, the liquid metal starts to flow in the opposite as shown in Figure 23e,f. With 1000 °C pre-heating the solidification is very slow which causes the melt pool more time to stabilize. Furthermore, as the flow moves backwards in Figure 23e,f and is still moving at 500 µs.

**Figure 24:**
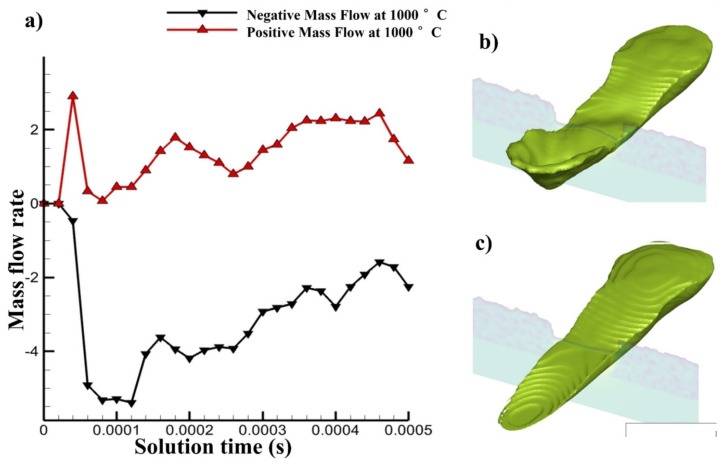
(**a**) Mass flow rate forward and backwards with 1000 °C pre-heating, (**b**,**c**) melt pool passing from cross section at different time.

At one point, the single track had a 2D cross section taken with 1000 °C pre-heating, the melt pool flowing forward and backward was calculated in Figure 24a through mass flow. It depicts the melt pool flow’s pattern before and after laser irradiation at 1000 °C pre-heating and compares the two. After being exposed to laser radiation, some of the melt pool flows ahead; however, when the laser passes across that location, some of the melt pool is dragged backwards. However, it keeps moving forward after the laser irradiation as shown in the graph, even though it also keeps moving backwards which is more significant than both previous ones. The cross section through which the mass flow has been described can be seen in Figure 24b,c.

**Figure 25:**
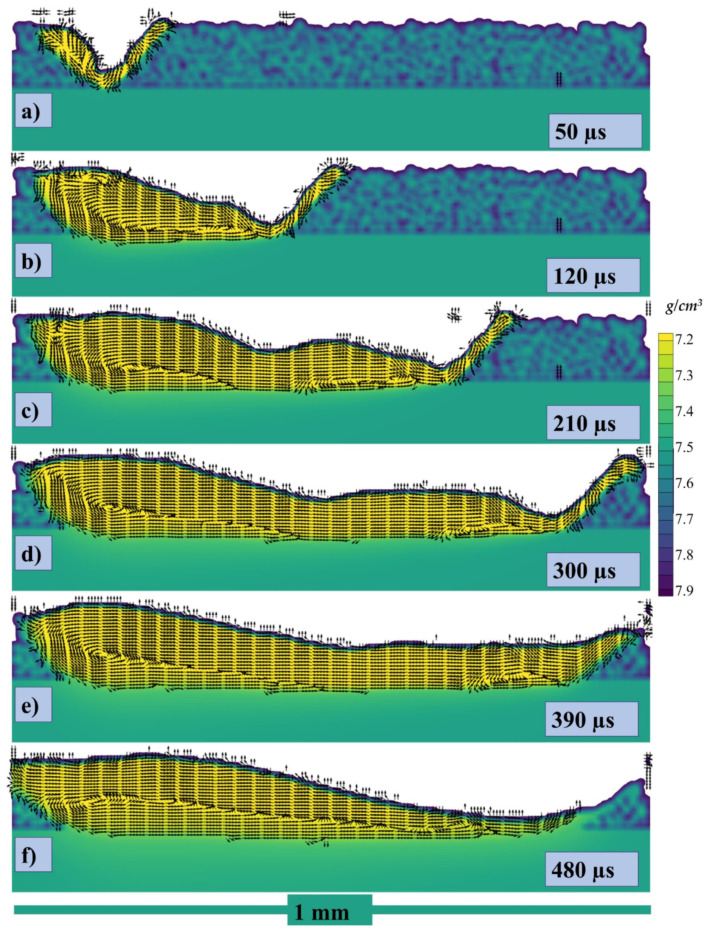
Velocity vectors of the cross section at (**a**) 50, (**b**) 120, (**c**) 210, (**d**) 300, (**e**) 390, (**f**) 480 µs.

The cross section with 1000 °C pre-heating has been taken with velocity vectors at each point within the melt pool and can be seen in Figure 25a–f at 50, 120, 210, 300, 390, and 480 µs, respectively. As in the example discussed above, when the laser starts to irradiate at 1000 °C the pre-heated powder particles start to form the melt pool more rapidly as shown in Figure 25a. A reaction force in the deeper depression zone is generated on the laser contacting surface as shown by the velocity vectors in the top surface in Figure 25a like the one at 500 °C pre-heating. However, as the laser progresses, due to the surrounding air the upper region of the melt pool which is in direct contact with the air starts to cool (can be visualized with the velocity vectors on the tope surface caused by Marangoni force as well as by the density color gradient) with huge increase in the surface tension, in Figure 25b–d.

A circular flow pattern can also be visualized with the velocity vectors at the rear end of the melt pool in Figure 25b–f. However, as the front-end temperature decreases with time and the surface tension over there increase, it starts to pull the liquid in the opposite direction as seen in Figure 25e,f from the bottom of the circular flow pattern.

**Figure 26:**
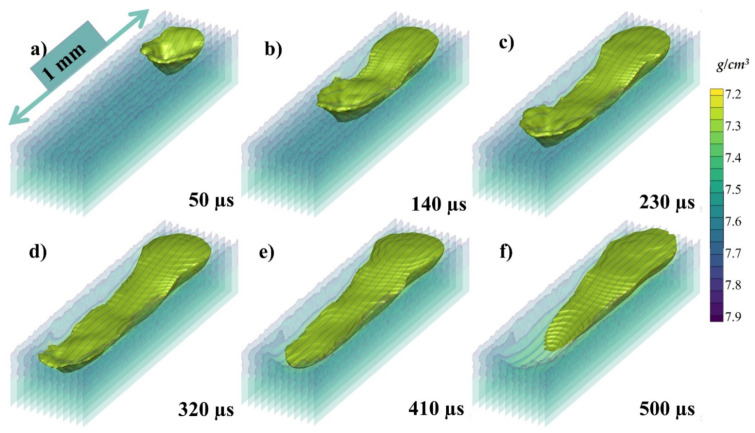
The isometric view of the melt pool with 1000 °C pre-heating (**a**) 50, (**b**) 120, (**c**) 230, (**d**) 320, (**e**) 410, (**f**) 500 µs.

The isometric view of the melt pool with 1000 °C pre-heating has been taken without the solid powder (transparent region) and can be seen in Figure 26 at 50, 120, 230, 320, 410, and 500 µs, respectively, similar to the one at 500 °C pre-heating in Figure 13. Here, the melt pool flow can be visualized which is wider and deeper with the same energy density. When the laser starts to irradiate a depression is formed as shown in Figure 26a and the recoil pressure and Marangoni pushes the melt pool backwards as shown in Figure 26b, and the surface tension furthers the pull as shown in Figure 26c. After the laser irradiation has been finished the dominating or the only force that is contributing to the flow is Marangoni force as shown in Figure 26d–f. The frames per micro-second in video format for (1) cross sectional stream traces at 1000 °C, (2) cross sections velocity vectors at 1000 °C, (3) velocity vectors isometric view at 1000 °C, and (4) velocity vectors top view at 1000 °C, can be obtained from Supplementary Data.

**Figure 27:**
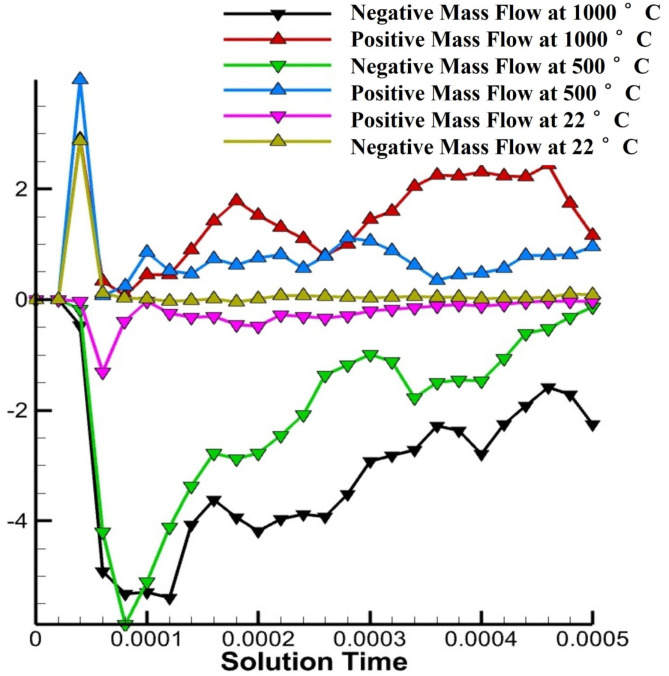
Mass flow rate forward and backwards from cross section at different time at RT in pink and yellow line, at 500 °C pre-heating in green and blue line and at 1000 °C pre-heating, in black and red line.

The mass flow pushed forward and pulled backward at RT, 500 °C and 1000 °C pre-heating has been shown Figure 27. Here, the change in the mass flow can be understood clearly. As we pre-heat the mass flow has more time to stabilize which leads to positive mass flow even after the laser irradiation. However, at the room temperature the forward mass flow is stopped after the irradiation.

## 4. Conclusions

We have exposed and measured the melt flow dynamics throughout the whole melt pool using velocity vectors at different points, stream traces of the fluid movement, and mass flow rate forward and backward movement. in the LPBF for the first time in this study. The main conclusions are discussed in depth below:

1. The melted flow patterns in every region of the entire melt pool have shown a fairly complicated flow pattern due to the interaction of several driving factors. We measured and derived equation a drop in flow velocity from the depression region to the melt pool rear region.
2. The propelling factors for various melt flow variations were studied. The Marangoni effect is responsible for the circulation flow from a low surface tension to a high surface tension region on the melted surface. The fluid movement all across depression-zone sidewalls is dominated by evaporation. Hydraulic pressure propels fluid movement from an area of high pressures to a low-pressure area. Buoyance pressure is responsible for fluid convection between low- and high-density regions.
3. Only at laser irradiating region of its melt pool, the powerful major factors are recoil liquid momentum and heat convection; just at rear of the melt pool, the dominant major factors have been determined for being high surface tension and the thermal conductivity, respectively.
4. Throughout this study, modeling shows an increase in pre-heating level across the melt pool variability (wide, height, and duration).
5. greater pre-heating temperature create a melt pool having greater depth and relatively dimension. In modeling, a front sidewall inclination of irradiating region increases with pre-heating temperatures, indicating more laser drill force. Increasing the temperature degree increases penetration.
6. As the solidification speed rises, the melting period tends to become shorter. Due to increased retribution force and fluid suppressing, the reversed melt flow from both the laser center region and Marangoni power is not possible leading to higher residual stresses. Lower pre levels typically have faster movement. At higher pre-heating levels the residual stresses can be reduced as the melt pool has more time to release the pressures.

A much more advanced in-situ monitoring system would aid in achieving more accurate heat transmission within the melting zone. The research contemplates even more comprehensive visual investigation into the development of melt pools associated variability changes using precise multi-physical simulations. This will help with the processing parameters guidelines, which are designed to prevent defects or greatly improve the productivity of the items manufactured by PBF processes. Future work may address other important processing parameter studies with respect to scanning speed and laser power with the Marangoni effects.

## Figures and Tables

**Table 1:** IN718 particle size (µm).

D10	D50	D90
19	29	41

**Table 2:** Chemical composition of In718.

Ni	Cr	Fe	Al	Co	Cu	Mn	Mo	Nb	Si	Ta	Ti	B	C
52.5	19.5	17.3	0.5	0.5	0.15	0.175	3.05	5.125	0.175	0.025	0.9	0.003	0.04

**Table 3:** Technical Specification ERMAKSAN Enavision 250.

GENERAL SPECIFICATION	ENAVISION 250
Production Volume (mm^3^)	250 × 250 × 300 (9.8 × 9.8 × 11.8 inch)
Adjustable Layer Height	20–100 μm (0.0007–0.004 inch)
Laser Type	Fiber Laser
Laser Power	500W
Scanning Speed	Up to 11 m/s (433.07 inch)
Scanning System	3D Dynamic Focused Scanning System
Dimension	(L × W × H) 2700 × 1440 × 2030 (106.3 × 56.7 × 79.9 inch)
Voltage	400 V, 3 PH, 50/60 Hz
Current	32 A
Inert Gas	Argon/Nitrogen
02 Level	100 ppm
Vacuum Pomp	Yes
Operating System	Operating SystemWindows 10/X
**CONTROL UNIT**	
Control System	Beckhoff Industrial PC
Processor	ProcessorIntel i5–i7
Operating System	Windows 10/X
HMI	15.6 inch, Touch Operated
**SOFTWARE**	
Data Preparation Software	Materilliase Magics and Modules
Data Processing Software	Ermaksan Build Processor
Supported File Types	STL, 3MF, AMF, DAE, FBX, VRML.

## Data Availability

Not applicable.

## Supplementary Materials

The following are available online at https://www.mdpi.com/article/10.3390/ma14216683/s1, Supplementary Data: Temperature Change, Supplementary Data: Density Change.
