# Supplementary figures and images for: Laser Powder Bed Fusion (LPBF) of In718 and the Impact of Pre-Heating at 500 and 1000 °C: Operando Study
Source: Materials (Basel). 2021 Nov 5;14(21):6683. doi: 10.3390/ma14216683 (PMC8588267; doi:10.3390/ma14216683)

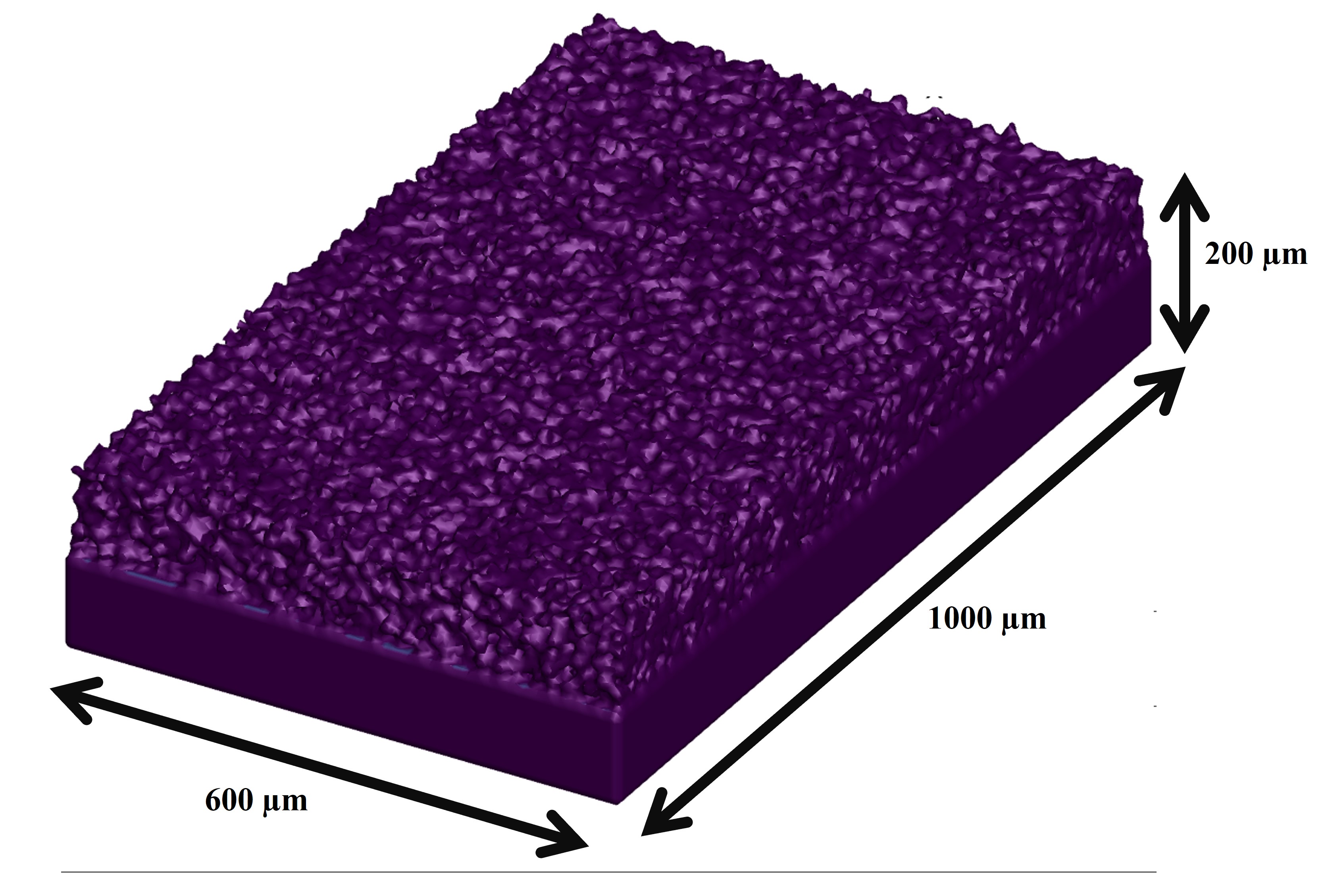

Supplement: Supplementary file 1 [file materials-14-06683-s001.zip › materials-1428908-supplementray.jpg]
